# Interfacial P-O-Cu Bonds Drive Rapid Z-Scheme Charge Transfer for Efficient Photocatalytic O_2_ Evolution Synchronized with Cr(VI) Reduction

**DOI:** 10.3390/nano15201592

**Published:** 2025-10-19

**Authors:** Yingcong Wei, Zeyu Su, Bo Weng

**Affiliations:** 1 School of Physics and Electronic Engineering, Jiangsu University, Zhenjiang 212013, China; ycwei@ujs.edu.cn (Y.W.);; 2 State Key Laboratory of Advanced Environmental Technology, Institute of Urban Environment Chinese Academy of Sciences, 1799 Jimei Road, Xiamen 361021, China; 3 University of Chinese Academy of Sciences, 19A Yuquan Road, Beijing 100049, China

**Keywords:** photocatalysis, P-O-Cu bonds, Z-scheme heterojunction, O_2_ evolution, Cr(VI) reduction

## Abstract

Addressing the challenges of energy production and environmental sustainability necessitates the development of advanced materials capable of facilitating both photocatalytic reduction and oxidation processes. Here, we report a Z-scheme Ag_3_PO_4_/CuBi_2_O_4_ heterojunction photocatalyst, which was fabricated via the in situ anisotropic growth of Ag_3_PO_4_ nanoparticles on the ends of CuBi_2_O_4_ microrods. The prepared heterojunction exhibits a low lattice mismatch (~3%) and features a covalently bonded interface, anchored by oxygen atoms, with the formation of P-O-Cu bonds. This interface synergizes with the built-in electric field to drive an efficient Z-scheme charge transfer mechanism, significantly enhancing the separation and migration of carriers. Furthermore, the interfacial chemical bonds induce electron redistribution that effectively weakens the Ag-O bond, thereby activating surface lattice oxygen. As a result, the photocatalyst shows remarkably improved performance for photocatalytic oxygen evolution synchronized with Cr(VI) reduction by enabling both the conventional adsorbate evolution mechanism and the lattice oxygen mechanism. This work provides critical insights into the design of efficient photocatalysts.

## 1. Introduction

Hexavalent chromium (Cr(VI)), identified as a highly soluble and carcinogenic heavy metal pollutant, is a prevalent contaminant in industrial effluents [1,2,3,4,5]. Its mutagenicity and bioaccumulation potential contribute to severe ecological and human health risks in aquatic environments [6,7]. Conventional methods like chemical reduction and adsorption face limitations, including secondary sludge generation, incomplete detoxification, and high operational costs [8,9]. Semiconductor photocatalysis has emerged as a highly attractive advanced oxidation/reduction technology for environmental remediation and solar energy conversion [10,11,12,13,14,15,16]. Current photocatalytic Cr(VI) reduction strategies require stoichiometric sacrificial electron donors (e.g., EDTA, methanol), which not only increase operational costs but also generate secondary pollutants, contradicting the principles of sustainable chemistry [3,17,18,19]. Synchronous coupling of Cr(VI) reduction with water oxidation to produce oxygen (O_2_) represents an ideal solution: 2H_2_O + Cr(VI) → O_2_ + Cr(III) + 4H^+^. However, achieving this requires the photocatalyst to simultaneously meet stringent thermodynamic and kinetic requirements. This includes possessing a conduction band potential sufficiently negative for Cr(VI) reduction and a valence band potential sufficiently positive for water oxidation. Furthermore, the catalyst must exhibit high electron–hole separation efficiency and provide ample reactive sites.

The Z-scheme heterojunction effectively preserves strong oxidation and reduction capabilities by mimicking natural photosynthesis and utilizing a unique Z-scheme charge transfer pathway [20,21,22]. The formation of a built-in electric field (BIEF) at the heterointerface is crucial for facilitating interfacial charge transfer [23,24,25]. Such a BIEF is readily induced by charge transfer mechanisms driven by diverse interactions utilized in heterojunction fabrication, such as hydrogen bonding [26,27], van der Waals forces [28,29], and electrostatic interactions [30,31,32,33,34]. However, the inherent instability of these interactions between constituent semiconductors frequently results in insufficient charge transfer. Heterojunction interfaces formed through chemical bonds, such as covalent, ionic, or coordination bonds, offer significant advantages over interfaces held together by simple physical contact or weak van der Waals forces [35,36,37,38,39,40]. On one hand, these strong chemical bonds provide robust interfacial adhesion, effectively resisting external stresses such as solution erosion and mechanical agitation, thereby preventing structural disintegration of the heterojunction. On the other hand, they establish continuous electronic pathways that lower interfacial energy barriers, significantly enhancing electron–hole transport across the interface [41,42,43,44]. Moreover, forming chemical bonds at the interface modifies the charge distribution, strengthening BIEF and further boosting charge separation efficiency. Therefore, designing a chemical bond-bridged heterointerface with a strong BIEF is highly desirable for synergistically optimizing photocatalytic Cr(VI) reduction coupled with O_2_ generation.

Herein, a P-O-Cu bond and IEF modulated Z-scheme heterojunction of Ag_3_PO_4_/CuBi_2_O_4_ with a small lattice mismatch is synthesized by in situ anisotropic growth of Ag_3_PO_4_ strategy on the end of the CuBi_2_O_4_ microrods for significantly enhanced photocatalytic O_2_ evolution and Cr(VI) reduction efficiency. Ag_3_PO_4_ and CuBi_2_O_4_ served as model materials due to interlaced band structures and distinct Fermi levels. An intimate covalent heterointerface was formed between Ag_3_PO_4_ and CuBi_2_O_4_ using the outermost O atoms as anchoring sites, resulting in a small interfacial mismatch of 3%. Systematic investigations reveal that the P-O-Cu bond and the IEF cooperatively induce the Z-scheme charge transfer mechanism, which in turn facilitates rapid charge separation and transfer [45,46]. More importantly, interfacial chemical bonding induces electron redistribution, significantly weakening the Ag-O bond energy and activating surface lattice oxygen. This, in turn, facilitates oxygen generation via the lattice oxygen pathway.

## 2. Materials and Methods

### 2.1. Chemicals

Sodium hydroxide (NaOH), bismuth nitrate pentahydrate (Bi(NO_3_)_3_·5H_2_O), dibasic sodium phosphate (Na_2_HPO_4_), copper nitrate trihydrate (Cu(NO_3_)_2_·3H_2_O), and silver nitrate (AgNO_3_) were obtained from Sinopharm Chemical Reagent Co., Ltd., Shanghai, China. These reagents were used without any treatment.

### 2.2. Synthesis of CuBi_2_O_4_ Microrods

CuBi_2_O_4_ microrods were synthesized by a one-step hydrothermal method. The specific process was as follows: First, 0.6 g of Cu(NO_3_)_2_·3H_2_O was sequentially dissolved in 80 mL of deionized water. Next, 2.42 g of Bi(NO_3_)_3_·5H_2_O and 0.87 g of NaOH were added to the above suspension under vigorous stirring, and stirred continuously at room temperature for 3 h. Finally, the resulting suspension was transferred to a 100 mL stainless steel autoclave and kept at 180 °C for 24 h of hydrothermal reaction. The final sample was washed several times in ionized water and ethanol, and was recorded as CuBi_2_O_4_ after drying at 70 °C for 12 h.

### 2.3. Synthesis of Ag_3_PO_4_/CuBi_2_O_4_ Composites

First, different contents of CuBi_2_O_4_ powder and 20 mL of deionized water were added to a beaker labeled as solution A. The sample was processed using an ultrasonic cell crusher for 1 h in order to achieve a uniform dispersion of the sample, which is conducive to obtaining CuBi_2_O_4_ microrods. Next, AgNO_3_ and Na_2_HPO_4_ were dissolved in 40 mL of deionized water, respectively, to obtain solution B and solution C. Subsequently, solution B was transferred to solution A, and solution C was instilled in a light-avoiding condition to change the color of the mixed solution from brown to bright yellow. Finally, the mixed solution was placed into a 100 mL Teflon-lined high-pressure cauldron and heated in an oven at 120 °C for 3 h. The precipitated product was washed with deionized water and anhydrous ethanol and dried under vacuum at 70 °C to obtain the desired yellow powder. The synthesized Ag_3_PO_4_/CuBi_2_O_4_ composite photocatalysts were named as ACBO-x, and x was 2, 5, 7, and 10, respectively (Here, x is the mass percentage of CuBi_2_O_4_ to Ag_3_PO_4_). Pure-phase Ag_3_PO_4_ nanoparticles were synthesized by the same method without the addition of CuBi_2_O_4_. The mass percentages of CuBi_2_O_4_ relative to Ag_3_PO_4_ in the ACBO-2, ACBO-5, ACBO-7, and ACBO-10 composite photocatalysts are 1.4%, 4.8%, 6.1%, and 8.9%, respectively, as determined by ICP analysis.

### 2.4. Characterization

The X-ray diffraction (D/MAX2500PC, Riga Corporation, Beveren, Belgium) patterns were used to determine the C phase structure of the catalysts. Field-emission scanning electron microscopy and energy spectroscopy were used for observation of material geometry, geometries, and dispersion states. The morphology was studied using a scanning electron microscope (SEM, FEI NovaNano450, FEI, Hillsboro, OR, USA) and a transmission electron microscope (TEM, JEM-2800, JEOL, Tokyo, Japan). The light absorption range of the photocatalyst was analyzed on a UV–visible near infrared spectrophotometer (UV-2600, Shimadzu Corporation, Kyoto, Japan). Electron spin resonance (ESR) experiments were carried out with a Bruker A 300 (Bruker Corporation, Ettlingen, Germany) spectrometer to determine the electron spin resonance phenomenon of unpaired electrons in matter. Fourier transform infrared spectroscopy (FTIR) (Nicolet iS50, Thermo Fisher Scientific, Waltham, MA, USA) was used to identify the functional groups present in the identified molecules with a KBr background and scanning range set to 400–4000 cm^−1^. Laser Raman spectroscopy (LRS) model VERTEX 80 Raman microscope (Bruker Corporation, Germany) was used to provide information on the chemical structure, phases, and morphology of the samples, crystallinity, and molecular interactions. The X-ray photoelectron spectroscopy (XPS) model ESCALAB 250XI (Thermo Fisher Scientific, Waltham, MA, USA) was used to analyze the elemental composition, chemical state, and molecular structure of the samples. Evaluation of photoluminescence efficiency of photocatalysts was carried out with a Quanmanster TM40 fluorescence spectrometer (PL, Photon Technology International, Birmingham, NJ, USA), and fluorescence lifetime of photocatalysts was studied by time-resolved fluorescence spectroscopy (TRPL)

### 2.5. Photoelectricity Experiments

The electrochemical performance of the samples was evaluated on an electrochemical workstation (CHI660E, Shanghai Chenhua Instruments, Shanghai, China) using Na_2_SO_4_ (0.5 mol/L) solution as the working electrolyte solution and Pt and Ag/AgCl electrodes as the counter electrode and reference electrode, respectively. For the preparation of the working electrodes, 5 mg of the sample, 250 μL of ethylene glycol, 250 μL of ethanol, and 40 μL of the membrane solution were mixed well and subsequently added dropwise onto a 1 × 2 cm FTO conductive glass with a coverage area of 1 cm^2^ of the sample. Subsequently, the sample was placed in a 70 °C oven and dried underneath for 4 h for the preparation of the working electrodes.

### 2.6. Photocatalytic Experiments

25 mg photocatalysts were dispersed in a beaker containing 50 mL of AgNO_3_ (0.6 M) solution, ultrasonically mixed homogeneously, and poured into a photocatalytic quartz reactor and evacuated for 15 min to maintain a sealed environment. During the reaction, the reaction system was maintained at 10 °C using circulating water and irradiated with a 300 W Xenon lamp equipped with a cutoff filter (λ ≥ 420 nm). Finally, gaseous products were analyzed using a Shimadzu GC-2014 gas chromatograph equipped with a thermal conductivity detector (TCD) and a molecular sieve column, using argon as the carrier gas. In the experiments using potassium dichromate as the sacrificial agent, basically the same test conditions were used. The difference was that an additional 4 mL of the reaction system solution sample was taken before and after illumination. The corresponding Cr(VI) conversion rate was calculated by comparing the absorption intensity of the UV–visible spectrophotometer at 532 nm. The calculation formula of the reduction conversion rate is R = (C_0_ − C/C_0_) × 100%, where R denotes the Cr(VI) reduction rate, C_0_ represents the concentration before the start of the reaction, and C denotes the concentration after the end of the reaction.

## 3. Results

### 3.1. Structural and Morphological Characterization

**Figure 1:**
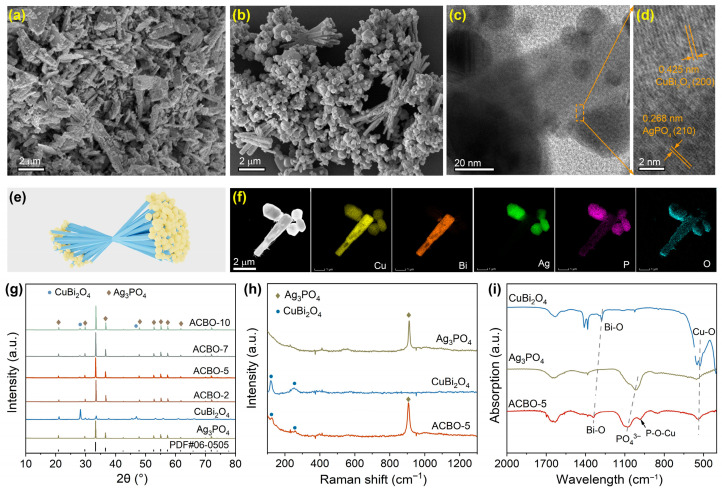
(**a**) SEM image of CuBi_2_O_4_. SEM (**b**), TEM (**c**), and HRTEM (**d**) images of ACBO-5. (**e**) Schematic diagram of the ACBO-5 sample morphology. (**f**) Elements mapping of Cu, Bi, Ag, P, and O in ACBO-5. XRD patterns (**g**), Raman spectra (**h**), and FTIR spectra (**i**) of the as-synthesized samples.

Synthesis of Ag_3_PO_4_/CuBi_2_O_4_ heterojunction was conducted in a two-step process. First, pristine CuBi_2_O_4_ microrods were synthesized through the hydrothermal method. Subsequently, Ag_3_PO_4_ was in situ anchored on the surface of CuBi_2_O_4_ via a facile hydrothermal route to craft Ag_3_PO_4_/CuBi_2_O_4_ composites. For simplicity, Ag_3_PO_4_/CuBi_2_O_4_ is denoted as ACBO. The morphology and microstructural characteristics of the as-prepared photocatalysts were analyzed by SEM and TEM images. Figure S1 displays irregularly spherical Ag_3_PO_4_ particles, exhibiting slight agglomeration. CuBi_2_O_4_ clearly demonstrates a one-dimensional (1D) microrod structure, where the microrods possess lengths of approximately 2–5 μm (Figure 1a). The SEM images of the ACBO-5 sample indicate a strong interfacial interaction along the central portions of the CuBi_2_O_4_ microrods, whereas their extremities show a tendency towards divergence. Notably, anisotropic growth of the Ag_3_PO_4_ particles was observed, with preferential material deposition occurring at one terminal end (Figure 1b,e). The tetragonal CuBi_2_O_4_ microrod attaches to the (200) plane of body-centered cubic Ag_3_PO_4_ through its (210) plane, and the growth direction of CuBi_2_O_4_ microrods is along the [100] direction (Figure 1c,d). The observed intimate attachment is crucial for lowering charge transport barriers, resulting in enhanced ultrafast interfacial charge transfer. EDX-STEM elemental mapping (Figure 1f) clearly shows that Cu and Bi are preferentially localized in the stem region of the rod, whereas Ag and P are mainly distributed at the ends of the rod-like structure. Oxygen (O) is uniformly distributed across the entire microrod.

The typical powder XRD patterns for the Ag_3_PO_4_, CuBi_2_O_4_, and ACBO-5 heterojunction with different amounts of Ag_3_PO_4_ are displayed in Figure 1g. The diffraction peaks of Ag_3_PO_4_ mainly located at 2θ = 20.8°, 29.7°, 33.3°, 36.6°, 46.8°, 52.7°, 55.0°, 57.3 °, and 71.9°, corresponding to the (110), (200), (210), (211), (310), (222), (320), (321), and (421) crystal planes of Ag_3_PO_4_ (JCPDS no. 06-0505), respectively [47]. For the pure CuBi_2_O_4_, four remarkable diffraction peaks were observed at 2θ = 21.0°, 28.2°, 33.4°, and 46.3°, corresponding to the (200), (131), (130), and (141) crystal planes of CuBi_2_O_4_ (JCPDS no. 720493), which were in agreement with the literature report [48]. It is worth noting that the diffraction peaks of the Ag_3_PO_4_/CuBi_2_O_4_ composite material can simultaneously exhibit the characteristic diffraction peaks of Ag_3_PO_4_ and CuBi_2_O_4_. As the CuBi_2_O_4_ content increased from 2 to 10%, the main characteristic peaks of the samples still existed, but the characteristic peaks at 28.2° gradually strengthened. All these XRD results confirmed that Ag_3_PO_4_ and CuBi_2_O_4_ successfully combined together. As shown in Figure 1h, the Raman spectra of CuBi_2_O_4_ were consistent with those reported in the literature, indicating the successful preparation of CuBi_2_O_4_. For the Raman spectra of Ag_3_PO_4_, the peak located at 909.8 cm^−1^ can be severally assigned to the symmetric stretching vibration of PO_4_^3−^ of Ag_3_PO_4_ (Figure 1i). The peaks at 121.2 cm^−1^ correspond to lattice vibrations involving the heavier Bi and Cu cations, while the peak at 253.6 cm^−1^ is associated with the stretching and contraction of the Bi-O bonds in CuBi_2_O_4_. The Raman spectra of the composite material displayed characteristic peaks corresponding to both Ag_3_PO_4_ and CuBi_2_O_4_. This observation provides strong corroboration for the successful formation of the Ag_3_PO_4_/CuBi_2_O_4_ heterojunction. This observation provides strong corroboration for the successful formation of the Ag_3_PO_4_/CuBi_2_O_4_ heterojunction, further supported by the complementary XRD analysis.

Figure 1i presents the Fourier transform infrared (FTIR) spectra of the as-obtained samples. All the samples showed a significant absorption peak at 1627 cm^−1^, which was caused by the bending vibration modes of the O-H groups of adsorbed water on the catalyst surface. For CuBi_2_O_4_, infrared absorption peaks were observed at 1398 cm^−1^ and 477 cm^−1^, corresponding to the characteristic vibrations of Bi-O and Cu-O bonds, respectively [49]. Furthermore, the Ag_3_PO_4_ materials exhibited an absorption peak at 1094 cm^−1^, which corresponds to the P=O stretching vibration mode of PO_4_^3−^ [50]. The P=O stretching vibration in the composites experienced a shift to a higher wavenumber relative to that of pristine Ag_3_PO_4_. This observation not only confirms the successful formation of the heterojunction but also suggests a significant interaction at the interface between CuBi_2_O_4_ and Ag_3_PO_4_. Notably, a new characteristic peak observed at 982 cm^−1^ in the composites can be indexed to a P-O-Cu bonding state [51], which provides direct evidence for the intimate interface between CuBi_2_O_4_ and Ag_3_PO_4_ formed through the creation of P-O-Cu bonds.

**Figure 2:**
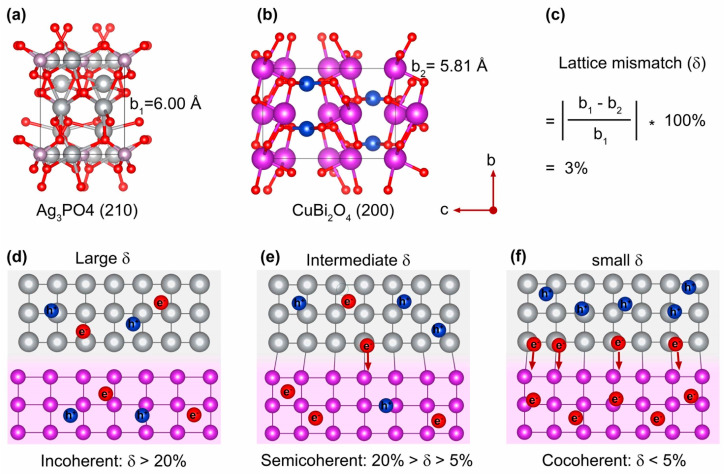
Crystal structures and lattice constants of Ag_3_PO_4_ (210) (**a**) and CuBi_2_O_4_ (200) (**b**). (**c**) The calculation of interfacial lattice mismatch. Schematic illustration of heterojunctions with large δ (**d**), intermediate δ (**e**), and small δ (**f**).

The lattice mismatch (δ) at the interface of two dissimilar semiconductors is a crucial, yet often underappreciated, factor that plays a significant role in determining photocatalytic performance [52,53]. The lattice constants for Ag_3_PO_4_ (210) and CuBi_2_O_4_ (200) are b1 = 6.00 Å (Figure 2a) and b2 = 5.81 Å (Figure 2b), respectively. This results in a δ value of only 3% between the Ag_3_PO_4_ and CuBi_2_O_4_ planes (Figure 2c). Heterointerfaces can be broadly categorized into three types based on the degree of δ: incoherent interfaces (δ > 20%, Figure 2d), semicoherent interfaces (5% ≤ δ ≤ 20%, Figure 2e), and coherent interfaces (δ < 5%, Figure 2f). Heterostructures with a large δ often hinder photocatalytic activity due to the formation of extensive grain boundaries, inefficient heterointerfaces, and slow charge carrier migration. In contrast, heterostructures with a small δ are considered optimal photocatalysts, as they minimize grain boundaries, promote effective heterointerfaces, and facilitate rapid charge transfer across the junction. The resulting Ag_3_PO_4_ and CuBi_2_O_4_ heterojunction, linked via a covalent P-O-Cu bridge, was thus meticulously engineered with a small interfacial lattice mismatch, which is well below the 5% threshold for a coherent interface. Theoretically, the proposed structure is designed to facilitate rapid charge transfer, which is expected to be the basis for enhanced photocatalytic performance.

### 3.2. Photocatalytic Activity of the Ag_3_PO_4_/CuBi_2_O_4_ Photocatalyst

**Figure 3:**
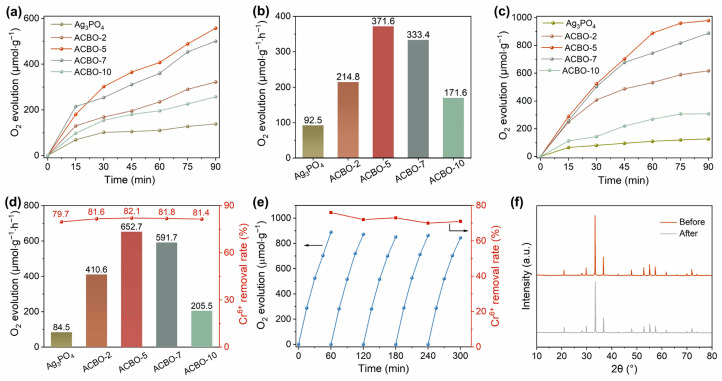
(**a**,**b**) Photocatalytic oxygen evolution performance of Ag_3_PO_4_ and Ag_3_PO_4_/CuBi_2_O_4_ composite with AgNO_3_ serving as an electron scavenger. (**c**,**d**) Photocatalytic performance of oxygen evolution synchronously with Cr(VI) reduction. (**e**) Cycling experiment of ACBO-5. (**f**) XRD patterns of ACBO-5 before and after the cyclic experiment.

In order to investigate the photocatalytic activity of Ag_3_PO_4_/CuBi_2_O_4_ composite, the water oxidation reaction was conducted under visible light irradiation. Figure 3a,b illustrate that pure Ag_3_PO_4_ displayed a modest O_2_ evolution rate of only 92.5 µmol·g^−1^·h^−1^, with AgNO_3_ serving as an electron scavenger. This limited activity is attributed to the facile recombination of photogenerated carriers. In comparison, a significant enhancement in photocatalytic O_2_ evolution activity was achieved through the incorporation of CuBi_2_O_4_. The O_2_ evolution rate of the ACBO composite was found to increase with the mass ratio of AgNO_3_ to CuBi_2_O_4_, reaching an optimal rate of up to 371.6 µmol·g^−1^·h^−1^ at a 5.0% AgNO_3_ loading. This represents an impressive enhancement, being approximately 4.1 times higher than that of pristine Ag_3_PO_4_. When potassium dichromate (K_2_Cr_2_O_7_) was employed as the electron scavenger instead of AgNO_3_, the photocatalytic O_2_ evolution rate was significantly enhanced (Figure 3c,d). The optimal ACBO-5 composite demonstrated robust photocatalytic activity, achieving an O_2_ evolution rate of 652.7 µmol·g^−1^·h^−1^ and a Cr(VI) reduction efficiency of 82.1%. The ACBO-5 demonstrates superior photocatalytic oxygen evolution activity, outperforming a range of reported catalysts (Table S2). This highlights the great potential of our design strategy for advancing solar-driven water splitting applications. The enhanced performance can be attributed to the rod-like structure of CuBi_2_O_4_, which minimizes charge migration pathways, drives efficient photoinduced carrier separation, and provides abundant surface active sites. Excessive loading of AgNO_3_ (e.g., in ACBO-7 and ACBO-9) was observed to be counterproductive for photocatalytic activity, primarily attributed to the excessive AgNO_3_ shielding the light absorption of CuBi_2_O_4_. The above results indicate that the loading of CuBi_2_O_4_ has a positive effect on enhancing the photocatalytic activity of Ag_3_PO_4_, and the ACBO composite is a catalyst with high photocatalytic reaction activity. The stability of a catalyst is a critical factor in determining its successful application in photocatalysis. It has been previously reported that the photocatalytic efficiency of Ag_3_PO_4_ is often compromised due to the favorable reduction of Ag^+^ to Ag^0^ nanoparticles [54,55]. While the ACBO-5 heterojunction system demonstrates expected photocatalytic activity, its long-term photochemical stability has yet to be fully established. To assess its long-term stability, cyclic photocatalytic tests were performed on ACBO-5. The photocatalytic activity of ACBO-5 remained stable, showing no significant decrease over five cycles (Figure 3e). The concentrations of Cu, Ag, and Bi ions in the solution were determined by ICP after the photocatalytic reaction (Table S3). The results show that the ion concentrations of Cu, Ag, and Bi in the solution were minimal, indicating that the material remains stable and that ion release is negligible under the tested conditions. As shown in Figure 3f, the consistent positions of the XRD diffraction peaks and the absence of any diffraction peaks corresponding to precipitated Ag particles before and after the reaction conclusively demonstrate that the ACBO-5 sample maintains high reactivity and stability throughout the photocatalytic synchronous reaction [56,57,58].

### 3.3. Insights into Z-Scheme Heterojunction Formation

**Figure 4:**
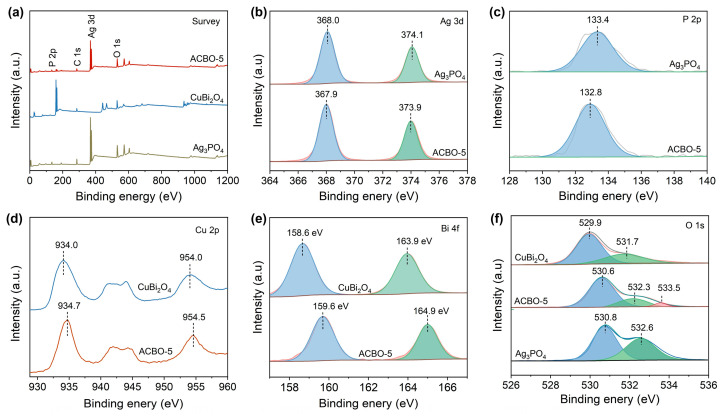
(**a**) Survey, (**b**) Ag 3d, (**c**) P 2p, (**d**) Cu 2p, (**e**) Bi 4f, and (**f**) O 1s XPS spectra.

The surface elemental compositions and corresponding chemical valence of Ag_3_PO_4_, CuBi_2_O_4_, and ACBO-5 were analyzed by XPS. The XPS survey spectrum of the ACBO-5 composite confirms the presence of Ag, P, O, Cu, and Bi elements, thereby providing evidence for the successful coupling of Ag_3_PO_4_ and CuBi_2_O_4_ (Figure 4a). As shown in Figure 4b, the Ag 3d XPS spectra for Ag_3_PO_4_ exhibit two characteristic peaks at 368.0 and 374.1 eV, which are characteristic of Ag 3d5/2 and Ag 3d3/2, respectively, indicating the Ag^+^ oxidation state. Whereas in the ACBO-5 sample, a slight negative shift of the Ag 3d5/2 and Ag 3d3/2 peaks to 367.9 eV and 373.9 eV, respectively, was observed. The main XPS peak of P 2p is located at 133.4 eV, corresponding to P-O (Figure 4c). Additionally, the ACBO-5 exhibits a more negative binding energy (BE) compared to the Ag_3_PO_4_. As depicted in Figure 4d, the Cu 2p core-level spectrum of monomeric CuBi_2_O_4_ shows characteristic peaks for Cu 2p1/2 and Cu 2p3/2 at 954.3 and 934.5 eV, respectively. The resulting spin-orbit splitting of 19.8 eV is consistent with the presence of the Cu^2+^. The other pair of peaks at 940.7 and 943.6 eV was assigned to the satellite peak of Cu^2+^. In the ACBO-5 sample, the BEs of the Cu 2p core levels showed a negative shift relative to those of CuBi_2_O_4_. As shown in Figure 4e, the Bi 4f XPS spectrum of CuBi_2_O_4_ reveals two characteristic peaks at 158.6 eV and 163.9 eV, respectively. These spectral features are consistent with the Bi 4f7/2 and Bi 4f5/2, respectively, unequivocally indicating the Bi^3+^ oxidation state. When coupled with Ag_3_PO_4_, the Bi 4f peaks were observed to shift to higher binding energies, specifically to 159.6 eV and 164.9 eV. The O 1s spectrum (Figure 4f) can be deconvoluted into three distinct components at binding energies of 530.6, 532.3, and 533.5 eV, which are assigned to lattice oxygen, surface hydroxyl/adsorbed oxygen species, and oxygen involved in interfacial P-O-Cu bonding, respectively [59,60,61]. Those results above suggest electron accumulation from CuBi_2_O_4_ on the surface of Ag_3_PO_4_. Moreover, the ACBO material exhibits a lower electron density at the Ag sites, resulting in a more ionic metal–oxygen bond, which may facilitate lattice oxygen activation.

### 3.4. Photophysical and Electrochemical Properties

**Figure 5:**
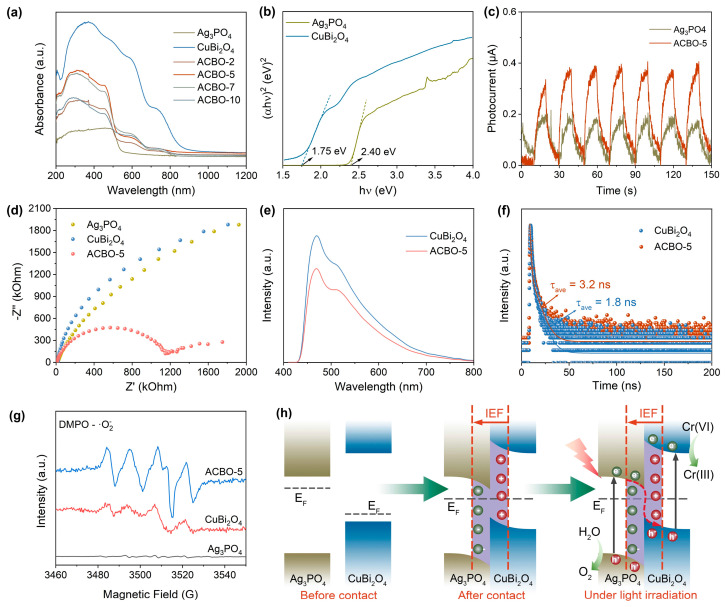
UV-vis DRS (**a**) of the prepared photocatalysts and the corresponding Tauc plots (**b**). Transient photocurrent curve density curves (**c**), EIS Nyquist plots (**d**), PL spectra (**e**), and TRPL spectra (**f**) of the different samples. (**g**) EPR spectra of DMPO-·O_2_^−^ over Ag_3_PO_4_, CuBi_2_O_4_, and ACBO-5. (**h**) The schematic of the Z-scheme heterojunction before and after hybridization, and under light illumination.

UV–visible diffuse reflectance spectroscopy (DRS) was employed to characterize the light absorption properties of the samples, as depicted in Figure 5a. The as-prepared Ag_3_PO_4_ samples displayed limited absorption in the visible light region, characterized by an absorption edge at 550 nm. The coupling of CuBi_2_O_4_ with Ag_3_PO_4_ resulted in a significant enhancement in the light absorption intensity of the ACBO heterostructure. The increased light absorption capability contributes to more efficient photocarrier generation, which is highly beneficial for improving photocatalytic performance. The band gaps of Ag_3_PO_4_ and CuBi_2_O_4_ were estimated to be 2.40 eV and 1.75 eV, respectively, using the Tauc equation (Figure 5b). Figure S2 illustrates the n-type semiconductor behavior of Ag_3_PO_4_ (positive slope) and the p-type semiconductor behavior of CuBi_2_O_4_ (negative slope). Their respective flat band potentials were determined to be −0.32 V and 0.82 V (vs. Ag/AgCl, pH = 6.8), respectively. The conduction band potentials for Ag_3_PO_4_ and CuBi_2_O_4_ are thus determined to be −0.27 V and −0.55 V (vs. NHE, pH = 0), respectively. The valence band positions (EVB) of Ag_3_PO_4_ and CuBi_2_O_4_ were subsequently derived as 2.67 V and 1.22 V (vs. NHE, pH = 0), respectively, by incorporating their determined band gap (Eg) into Eg = E_VB_ − E_CB_.

To further clarify the transfer pathway of photogenerated electrons, EPR spectroscopy was employed under illumination to capture superoxide (·O_2_^−^) radicals using 5,5-dimethyl-1-pyrroline N-oxide (DMPO) as a spin-trapping agent. As depicted in Figure 5g, significant DMPO-·O_2_^−^ signals are detected for CuBi_2_O_4_, whereas no such signals are observed for Ag_3_PO_4_. This difference is attributed to the relative positions of their conduction bands (CB) with respect to the standard reduction potential of O_2_/O_2_^−^ (−0.33 V vs. NHE). The CB of CuBi_2_O_4_ is sufficiently negative to reduce adsorbed O_2_ to ·O_2_^−^. Conversely, the CB of Ag_3_PO_4_ is significantly positive, preventing this reduction reaction from occurring. Compared to CuBi_2_O_4_, ACBO-5 exhibits a significantly stronger DMPO-·O_2_^−^ signal, indicating a more efficient accumulation of photogenerated electrons on the surface of CuBi_2_O_4_ under irradiation. Those results further provide strong evidence that the photogenerated charge transfer in the ACBO heterojunction follows a Z-scheme mechanism.

Based on the aforementioned characterization results, the photocatalytic reaction mechanism of ACBO can be displayed in Figure 5h. Upon contact between the two materials, free electrons from CuBi_2_O_4_ spontaneously migrate to Ag_3_PO_4_ until their Fermi energy levels equilibrate. This results in downward band bending at the Ag_3_PO_4_ interface (due to electron aggregation) and upward band bending at the CuBi_2_O_4_ (due to electron depletion). Consequently, a built-in electric field (IEF) pointing from CuBi_2_O_4_ to Ag_3_PO_4_ was established, thereby preventing further electron transfer between the materials. Under illumination, electrons within both Ag_3_PO_4_ and CuBi_2_O_4_ undergo excitation, promoting their transition from the VB to the CB. Consequently, under the influence of the IEF, energy band bending, and Coulombic attraction, the photogenerated electrons in Ag_3_PO_4_ preferentially recombined with photogenerated holes in CuBi_2_O_4_. This process preserves photo-induced carriers exhibiting the highest redox potential, which then contribute to subsequent photocatalytic reactions.

To better understand the high photocatalytic activity of the ACBO-5 heterojunction system, we employed transient photocurrent response, electrochemical impedance spectroscopy (EIS), photoluminescence (PL), and time-resolved photoluminescence (TRPL) to investigate the separation and transfer characteristics of photogenerated charge carriers. In the photocurrent test (Figure 5c), the photoresponsivity of ACBO-5 was significantly higher than that of the individual monomers, Ag_3_PO_4_ and CuBi_2_O_4_. This compelling result unequivocally demonstrates that the ACBO-5 composite photocatalyst possesses superior internal and interfacial separation efficiency of photogenerated charge carriers, which directly contributes to its excellent photocatalytic activity [62,63]. Figure 5d presents the Nyquist curve obtained from the EIS analysis of the samples. The charge transfer resistance, as indicated by the semicircle radii, decreased in the order CuBi_2_O_4_ > Ag_3_PO_4_ > ACBO-5, suggesting that the interfacial charge transfer is significantly accelerated upon the binding of Ag_3_PO_4_ to CuBi_2_O_4_. Furthermore, a substantial decrease in the PL emission intensity was observed for the ACBO-5 photocatalyst relative to pristine CuBi_2_O_4_, indicating that the heterojunction formation plays a crucial role in suppressing carrier recombination (Figure 5e). In addition, TRPL decay spectra revealed that ACBO-5 exhibited a longer average lifetime compared with that of pure CuBi_2_O_4_, indicating that the fabricated ACBO heterojunction facilitates more efficient charge separation (Figure 5f).

### 3.5. Mechanism of Light-Driven O_2_ Evolution Coupled with Cr(VI) Reduction

**Figure 6:**
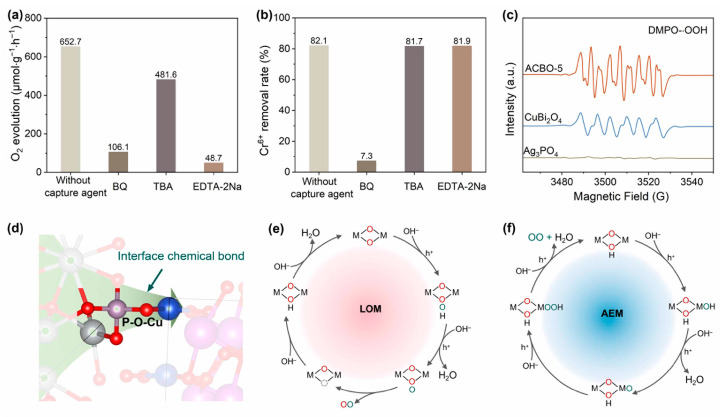
Photocatalytic O_2_ evolution (**a**) synchronously with Cr(VI) reduction (**b**) performance of ACBO-5 under various experimental conditions. (**c**) In situ EPR spectra of DMPO-·OOH. (**d**) Schematic diagram of the P-O-Cu interfacial bond formation. Schematic illustration of the LOM (**e**) and AEM (**f**) pathway on ACBO-5.

To gain in-depth insights into the photocatalytic reaction mechanism of ACBO-5, a series of free radical capture experiments was conducted (Figure 6a,b). 1,4-benzoquinone (BQ), Tert-butyl alcohol (TBA), and ethylenediaminetetraacetic acid disodium (EDTA-2Na) were employed as capture agents for ·O^2−^, ·OH, and h^+^, respectively. The introduction of BQ significantly suppressed the photocatalytic activity of ACBO-5. Specifically, the oxygen evolution rate decreased from 652.7 µmol·g^−1^·h^−1^ to 106.1 µmol·g^−1^·h^−1^, while the corresponding Cr(VI) reduction efficiency fell from 82.1% to merely 7.3%. Given that BQ (E0 ≈ +0.1 V vs. NHE) exhibits a much higher electron capture efficiency than Cr(VI), the reduction efficiency of Cr(VI) consequently decreased significantly due to this competition. The semiquinone free radicals (BQ·), generated by BQ capturing electrons, can further consume crucial reactive oxygen intermediates, peroxyl radicals (·OOH). This consumption interrupts the four-electron reaction pathway of OER, leading to a significant deterioration in OER performance. Upon the addition of TBA as a scavenger, the oxygen evolution activity of the composite photocatalyst was reduced by 26%, indicating that the formation of ·OH is a significant, but not dominant, step in the photocatalytic oxidation pathways. These findings indiscreetly demonstrate the direct involvement of surface lattice oxygen in the O_2_ evolution reaction, with a remarkable contribution rate of about 84%. Additionally, the O_2_ evolution activity significantly dropped to 48.7 µmol·g^−1^·h^−1^ after the addition of EDTA-2Na scavenger, indicating the crucial role of h^+^ in the photocatalytic water oxidation process. Conversely, the Cr(VI) reduction efficiency remained largely unaffected by the addition of either EDTA-2Na or TBA, consistently staying above 80%, suggesting a negligible role for ·OH and h^+^ in driving the Cr(VI) reduction process [64].

To gain deeper insights into the mechanism of light-driven O_2_ evolution, in situ EPR was employed to monitor the intermediates (Figure 6c). The photocatalytic systems of CuBi_2_O_4_ and ACBO-5 exhibited characteristic ·OOH radical peaks; however, these peaks were absent in the Ag_3_PO_4_ systems, which is consistent with the radical quenching results. Quantitative analysis revealed a 1.4-fold amplification of DMPO-·OOH adduct signals in the ACBO-5 system compared to pure CuBi_2_O_4_. These findings underscore the enhanced ·OOH generation driven by the heterojunction formation, which is consistent with the results of activity tests.

Based on the results above, two mainstream reaction pathways are involved in the oxygen evolution reaction (OER) mechanism: the lattice oxygen mechanism (LOM, Figure 6e) and the adsorbate evolution mechanism (AEM, Figure 6f). The earlier mechanism dominates under these conditions. The formation of interfacial chemical bonds serves a dual role: it enhances charge carrier separation while also inducing a rearrangement of interfacial electrons that consequently weakens the Ag-O bond, which in turn promotes increased lattice oxygen activity (Figure 6d). Consequently, the rate of oxygen generation increases via a mechanism that facilitates lattice oxygen participation.

## 4. Conclusions

We successfully fabricated an innovative Ag_3_PO_4_/CuBi_2_O_4_ composite, in which Ag_3_PO_4_ nanoparticles are loaded onto one end of CuBi_2_O_4_ microrods, synthesized through an in situ anisotropic growth strategy. This composite features a tightly bonded covalent heterointerface formed by P-O-Cu bonds, which synergistically interacts with the internal electric field (IEF) to induce a Z-type charge transfer mechanism. This unique charge flow dramatically improves charge separation and migration, leading to significant enhancements in both Cr(VI) reduction and oxygen generation. Furthermore, our analysis reveals that the interfacial chemical bonding triggers electron redistribution, which reduces the Ag-O bond energy and activates surface lattice oxygen. This activation is key to accelerating oxygen evolution through a highly efficient lattice oxygen pathway.

## Data Availability

The data supporting the findings of this study are available within the article and its Supplementary Materials. Additional raw data are available from the corresponding author upon reasonable request.

## Supplementary Materials

The following supporting information can be downloaded at: https://www.mdpi.com/article/10.3390/nano15201592/s1, Figure S1: SEM image of Ag_3_PO_4_; Figure S2: Mott-Schottky (M-S) plot of (a) Ag_3_PO_4_ and (b) CuBi_2_O_4_. Table S1. Mass ratio of CuBi_2_O_4_ to Ag_3_PO_4_ in the composite photocatalyst determined by ICP analysis. Table S2. Comparison of the photocatalytic oxygen evolution performance. Table S3. Concentration of Cu, Ag, and Bi ions in solution determined by ICP after photocatalytic reaction.

## References

[1] Duan L., Cheng T., Zhu Y., Wang Y., Gao Y., Bi J. (2025). Lanthanide-Porphyrin MOF as a Multifunctional Platform for Detection and Integrated Elimination of Cr(VI) and Ciprofloxacin. Inorg. Chem..

[2] Li X., Liu R.H., Han X.K., Ma X.X., Zhang L., Zhu H.J., Kong X.J., Li X., Yan H., Zhou H.W. (2024). Enhancing Photoreduction of Cr(VI) through a Multivalent Manganese(II)-Organic Framework Incorporating Anthracene Moieties. Inorg. Chem..

[3] Liu H., Li P., Zhang T., Zhu Y., Qiu F. (2020). Fabrication of Recyclable Magnetic Double-Base Aerogel with Waste Bioresource Bagasse as the Source of Fiber for the Enhanced Removal of Chromium Ions from Aqueous Solution. Food Bioprocess..

[4] Li L., Liu G., Dong J., Zhang Y., Cao S., Wang K., Wang B., She Y., Xia J., Li H. (2024). In Situ Construction of CuTCPP/Bi_4_O_5_Br_2_ Hybrids for Improved Photocatalytic CO_2_ and Cr(VI) Reduction. Inorg. Chem..

[5] Yin L., Jayan H., Cai J., El-Seedi H.R., Guo Z., Zou X. (2023). Development of a Sensitive SERS Method for Label-Free Detection of Hexavalent Chromium in Tea Using Carbimazole Redox Reaction. Foods.

[6] Qing Y., Gao W., Long Y., Kang Y., Xu C. (2023). Functionalized Titanium-Based MOF for Cr(VI) Removal from Wastewater. Inorg. Chem..

[7] Wang J., Ahmad W., Hassan M.M., Zareef M., Viswadevarayalu A., Arslan M., Li H., Chen Q. (2020). Landing Microextraction Sediment Phase onto Surface Enhanced Raman Scattering to Enhance Sensitivity and Selectivity for Chromium Speciation in Food and Environmental Samples. Food Chem..

[8] Buccato D.G., Ullah H., De Lellis L.F., Morone M.V., Larsen D.S., Di Minno A., Cordara M., Piccinocchi R., Baldi A., Greco A. (2024). Efficacy and Tolerability of a Food Supplement Based on Zea mays L., Gymnema sylvestre (Retz.) R. br. ex Sm, Zinc and Chromium for the Maintenance of Normal Carbohydrate Metabolism: A Monocentric, Randomized, Double-Blind, Placebo-Controlled Clinical Trial. Nutrients.

[9] Han S., Xie H., Zhang L., Wang X., Zhong Y., Shen Y., Wang H., Hao C. (2023). High-Performance Polyethylenimine-Functionalized Lignin/Silica Porous Composite Microsphere for the Removal of Hexavalent Chromium, Phosphate, and Congo Red from Aqueous Solutions. Ind. Crop. Prod..

[10] Yang L., Yuan Z., He L., Han L., Li B., Xu Y. (2024). Polyoxometalate Functionalized Cyclic Trinuclear Copper Compounds for Bifunctional Electrochemical Detection and Photocatalytic Reduction of Cr(VI). Inorg. Chem..

[11] Du Y., Li Y., Huang G., Pu H., Li Q., Lu C., Tan L., Dong L., Zhou C. (2024). CdBi_2_S_4_-Decorated Aminated Polyacrylonitrile Nanofiber for Photocatalytic Treatment of Cr(VI) and Tetracycline Wastewater. Inorg. Chem..

[12] Ghaedrahmati L., Jafarzadeh M. (2025). One-Pot Synthesis of a CoS_x_@Ag_2_S/CdS Z-Scheme Photocatalyst for RhB Photodegradation and Cr(VI) Photoreduction. Langmuir.

[13] Xu J., Zhang X., Yan W., Xie T., Chen Y., Wei Y. (2024). Optimizing Electronic Density at Active W Sites for Boosting Photocatalytic H_2_ Evolution. Inorg. Chem..

[14] Costantino F., Kamat P.V. (2021). Do sacrificial donors donate H_2_ in photocatalysis?. ACS Energy Lett..

[15] Rajeshwar K., Thomas A., Janáky C. (2015). Photocatalytic activity of inorganic semiconductor surfaces: Myths, hype, and reality. J. Phys. Chem. Lett..

[16] Weng B., Zhang M., Lin Y., Yang J., Lv J., Han N., Xie J., Jia H., Su B.-L., Roeffaers M. (2025). Photo-assisted technologies for environmental remediation. Nat. Rev. Clean Technol..

[17] Santarcangelo C., Baldi A., Ciampaglia R., Dacrema M., Di Minno A., Pizzamiglio V., Daglia M. (2022). Long-Aged Parmigiano Reggiano PDO: Trace Element Determination Targeted to Health. Foods.

[18] Ahmed S., Razzaq I., Sardar R., Ahmad M.N., Aziz T., Shami A., Lin L. (2025). Food Security through Triacontanol Priming: Mitigating Chromium Stress and Boosting Yield in *Raphanus sativus* L.. Ital. J. Food Sci..

[19] Zarekarizi F., Ghasempour H., Habibi B., Morsali A., Ramazani A. (2024). Development of a Novel Mixed-Metal-Organic Framework: An Innovative Photocatalyst for Simultaneous Cr(VI) Reduction and Phenol Degradation. Inorg. Chem..

[20] Shi M., Wu X., Zhao Y., Li R., Li C. (2025). Unlocking the Key to Photocatalytic Hydrogen Production Using Electronic Mediators for Z-Scheme Water Splitting. J. Am. Chem. Soc..

[21] Song W., Chong K.C., Qi G., Xiao Y., Chen G., Li B., Tang Y., Zhang X., Yao Y., Lin Z. (2024). Unraveling the Transformation from Type-II to Z-Scheme in Perovskite-Based Heterostructures for Enhanced Photocatalytic CO_2_ Reduction. J. Am. Chem. Soc..

[22] Zhao D., Wang Y., Dong C.L., Huang Y.C., Chen J., Xue F., Shen S., Guo L. (2021). Boron-Doped Nitrogen-Deficient Carbon Nitride-Based Z-Scheme Heterostructures for Photocatalytic Overall Water Splitting. Nat. Energy.

[23] Wang Y., Shang X., Shen J., Zhang Z., Wang D., Lin J., Wu J.C.S., Fu X., Wang X., Li C. (2020). Direct and Indirect Z-Scheme Heterostructure-Coupled Photosystem Enabling Cooperation of CO_2_ Reduction and H_2_O Oxidation. Nat. Commun..

[24] Liu X., Zhao Y.M., Zhang X., Wang L., Shen J., Zhou M., Shen L. (2023). Data-Driven Discovery of Transition Metal Dichalcogenide-Based Z-Scheme Photocatalytic Heterostructures. ACS Catal..

[25] Li N., Shi M., Sun G., Wu M., Li Q., Shen W., Ma J. (2023). Z-Scheme CdIn_2_S_4_/Bi_2_WO_6_ Heterojunction for High Piezo-Photo Synergetic Performance. Inorg. Chem..

[26] Sun R., Zhu Z., Tian N., Zhang Y., Huang H. (2024). Hydrogen Bonds and In Situ Photoinduced Metallic Bi^0^/Ni^0^ Accelerating Z-Scheme Charge Transfer of BiOBr@NiFe-LDH for Highly Efficient Photocatalysis. Angew. Chem. Int. Ed..

[27] Wang L., Liu Y., Lin Y., Zhang X., Yu Y., Zhang R. (2022). Z-Scheme Cu_2_(OH)_3_F Nanosheets-Decorated 3D Bi_2_WO_6_ Heterojunction with an Intimate Hetero-Surface Contact through a Hydrogen Bond for Enhanced Photoinduced Charge Separation and Transfer. Chem. Eng. J..

[28] Wang L., Zheng X., Chen L., Xiong Y., Xu H. (2018). Van der Waals Heterostructures Comprised of Ultrathin Polymer Nanosheets for Efficient Z-Scheme Overall Water Splitting. Angew. Chem. Int. Ed..

[29] Yang T., Zheng B., Wang Z., Xu T., Pan C., Zou J., Zhang X., Qi Z., Liu H., Feng Y. (2017). Van der Waals Epitaxial Growth and Optoelectronics of Large-Scale WSe_2_/SnS_2_ Vertical Bilayer p-n Junctions. Nat. Commun..

[30] Canossa S., Wuttke S. (2020). Functionalization chemistry of porous materials. Adv. Funct. Mater..

[31] Zellner R. (2015). Biological responses to nanoscale particles Beilstein. J. Nanotechnol..

[32] Yu Q., Sun S., Puente-Santiago A.R., Wu C., Weng B. (2025). Zinc mediated electronic structure of CoP toward photocatalytic H_2_ evolution. Appl. Catal. B Environ. Energy.

[33] Zhou X., Wang T., He D., Chen P., Liu H., Lv H., Wu H., Su D., Pang H., Wang C. (2024). Efficient Photocatalytic Desulfurization in Air through Improved Photogenerated Carriers Separation in MIL-101/Carbon Dots-g-C_3_N_4_ Nanocomposites. Angew. Chem..

[34] Xing C., Yu G., Zhou J., Liu Q., Chen T., Liu H., Li X. (2022). Solar Energy-Driven Upcycling of Plastic Waste on Direct Z-Scheme Heterostructure of V-Substituted Phosphomolybdic Acid/g-C_3_N_4_ Nanosheets. Appl. Catal. B Environ..

[35] Yue H., Guo Z., Zhou Z., Zhang X., Guo W., Zhen S., Wang P., Wang K., Yuan W. (2024). S-S Bond Strategy at Sulfide Heterointerface: Reversing Charge Transfer and Constructing Hydrogen Spillover for Boosted Hydrogen Evolution. Angew. Chem. Int. Ed..

[36] Zhao Z., Wang Z., Zhang J., Shao C., Dai K., Fan K., Liang C. (2023). Interfacial Chemical Bond and Oxygen Vacancy-Enhanced In_2_O_3_/CdSe-DETA S-Scheme Heterojunction for Photocatalytic CO_2_ Conversion. Adv. Funct. Mater..

[37] Wang Y., Hu Z., Wang W., Li Y., He H., Deng L., Zhang Y., Huang J., Zhao N., Yu G. (2023). Rational Design of Defect Metal Oxide/Covalent Organic Frameworks Z-Scheme Heterojunction for Photoreduction CO_2_ to CO. Appl. Catal. B Environ..

[38] Zhu Z., Huang H., Liu L., Chen F., Tian N., Zhang Y., Yu H. (2022). Chemically Bonded α-Fe_2_O_3_/Bi_4_MO_8_Cl Dot-on-Plate Z-Scheme Junction with Strong Internal Electric Field for Selective Photo-Oxidation of Aromatic Alcohols. Angew. Chem. Int. Ed..

[39] Ono L.K., Park N.G., Zhu K., Huang W., Qi Y. (2017). Perovskite Solar Cells—Towards Commercialization. ACS Energy Lett..

[40] Zheng K., Pullerits T. (2019). Two dimensions are better for perovskites. J. Phys. Chem. Lett..

[41] Seigneur N., Mayer K.U., Steefel C.I. (2019). Reactive transport in evolving porous media. Rev. Mineral. Geochem..

[42] Lei J., Yang H., Weng B., Zheng Y.M., Chen S., Menezes P.W., Meng S. (2025). Optimization of adsorption sites for selective hydrobenzoin and syngas production in a single photoredox cycle. Adv. Energy Mater..

[43] Xia X., Pan J.H., Pan X., Hu L., Yao J., Ding Y., Wang D., Ye J., Dai S. (2019). Photochemical conversion and storage of solar energy. ACS Energy Lett..

[44] Li S., Meng S., Zhang H., Puente-Santiago A.R., Wang Z., Chen S., Muñoz-Batista M.J., Zheng Y.M., Weng B. (2025). Tailoring Redox Active Sites with Dual-Interfacial Electric Fields for Concurrent Photocatalytic Biomass Valorization and H_2_ Production. Adv. Funct. Mater..

[45] Zou Y., Yan Y., Xue Q., Zhang C., Bao T., Zhang X., Yuan L., Qiao S., Song L., Zou J. (2024). MOF-on-MOF Heterostructured Electrocatalysts for Efficient Nitrate Reduction to Ammonia. Angew. Chem. Int. Ed..

[46] He B., Xiao P., Wan S., Zhang J., Chen T., Zhang L., Yu J. (2023). Rapid Charge Transfer Endowed by Interfacial Ni-O Bonding in S-Scheme Heterojunction for Efficient Photocatalytic H_2_ and Imine Production. Angew. Chem. Int. Ed..

[47] Liu X.Q., Chen W.J., Jiang H. (2017). Facile Synthesis of Ag/Ag_3_PO_4_/AMB Composite with Improved Photocatalytic Performance. Chem. Eng. J..

[48] Gudipati N.S., Ramesh A., Vanjari S., Duvvuri S., Challapalli S. (2023). MnO_2_ and CuBi_2_O_4_ Hybrid Microstructures for Efficient Nonenzymatic Hydroxylamine Detection. J. Chem. Sci..

[49] Tortorelli M., Chakarova K., Lisi L., Hadjiivanov K. (2014). Disproportionation of Associated Cu^2+^ Sites in Cu-ZSM-5 to Cu^+^ and Cu^3+^ and FTIR Detection of Cu^3+^(NO)_x_ (x = 1,2) Species. J. Catal..

[50] Liu H.S., Chin T.S., Yung S.W. (1997). FTIR and XPS Studies of Low-Melting PbO-ZnO-P_2_O_2_ Glasses. Mater. Chem. Phys..

[51] Sang Y., Song G., Gao Z., Zhang X., Wang T., Li Y., Zhang L., Guo L. (2020). Sea Urchin-Like CuO Particles Prepared Using Cu_3_(PO_4_)_2_ Flowers as Precursor for High-Performance Ethanol Sensing. Nanotechnology.

[52] Hu J., Li B., Li X., Yang T., Yang X., Qu J., Yang H., Lin Z. (2024). Lattice Match-Enabled Covalent Heterointerfaces with Built-in Electric Field for Efficient Hydrogen Peroxide Photosynthesis. Adv. Mater..

[53] Liu J., Zhang J. (2020). Nanointerface Chemistry: Lattice-Mismatch-Directed Synthesis and Application of Hybrid Nanocrystals. Chem. Rev..

[54] Pang F., Liu X., He M., Ge J. (2015). Ag_3_PO_4_ Colloidal Nanocrystal Clusters with Controllable Shape and Superior Photocatalytic Activity. Nano Res..

[55] Liu Y., Fang L., Lu H., Li Y., Hu C., Yu H. (2012). One-Pot Pyridine-Assisted Synthesis of Visible-Light-Driven Photocatalyst Ag/Ag_3_PO_4_. Appl. Catal. B Environ..

[56] Chen L., Wang C., Wang Z., Li G. (2024). Pivotal Role of Water Vapor–Mediated Defect Engineering on SrTiO_3_ Nanofiber Toward Efficient Photocatalytic Water Splitting. Mater. Today Energy.

[57] Lv H., Suo Z., Zhang F., Wan B., Zhou C., Ng X., Wang G., Chen Y., Liu Y. (2025). Electronic Structure Engineering and a Cascade Electron Transfer Channel in a Ni_2_P/1T-WS_2_/ZnIn_2_S_4_ Ternary Heterojunction for Enhanced Photocatalytic Hydrogen Evolution: Construction, Kinetics, and Mechanistic Insights. Inorg. Chem. Front..

[58] Huang K., Feng B., Wen X., Hao L., Xu D., Liang G., Shen R., Li X. (2023). Effective Photocatalytic Hydrogen Evolution by Ti_3_C_2_-Modified CdS Synergized with N-Doped C-Coated Cu_2_O in S-Scheme Heterojunctions. Chin. J. Struct. Chem..

[59] Iatsunskyi I., Gottardi G., Micheli V., Canteri R., Coy E., Bechelany M. (2021). Atomic Layer Deposition of Palladium Coated TiO_2_/Si Nanopillars: ToF-SIMS, AES and XPS Characterization Study. Appl. Surf. Sci..

[60] Maslakov K.I., Teterin Y.A., Stefanovsky S.V., Kalmykov S.N., Teterin A.Y., Ivanov K.E. (2017). XPS Study of Uranium-Containing Sodium-Aluminum-Iron-Phosphate Glasses. J. Alloys Compd..

[61] Abouelnaga A.M. (2025). Enhanced UV–Visible Optical Response of Cu^2+^-Doped ZnAl_2_O_4_ Spinel Thin Films. J. Solid State Sci. Technol..

[62] Gopannagari M., Reddy K.A.J., Inae S., Bae H.S., Lee J., Woo T.G., Rangappa A.P., Kumar D.P., Reddy D.A., Kim T.K. (2023). High-Performance Silver-Doped Porous CuBi_2_O_4_ Photocathode Integrated with NiO Hole-Selective Layer for Improved Photoelectrochemical Water Splitting. Adv. Sustain. Syst..

[63] Zhang Z., Hao H., Yang H., Zhu L., Ding C., Zhang G., Bi J., Yan S., Liu G., Hou H. (2021). UV-Vis-NIR-Light-Driven Ag_2_O/Ag_2_S/CuBi_2_O_4_ Double Z-Scheme Configuration for Enhanced Photocatalytic Applications. Mater. Sci. Semicond. Process..

[64] Yang X., Wang Y., Xu X., Qu Y., Ding X., Chen H. (2017). Surface Plasmon Resonance-Induced Visible-Light Photocatalytic Performance of Silver/Silver Molybdate Composites. Chin. J. Catal..

