# Supplementary material for: Interfacial P-O-Cu Bonds Drive Rapid Z-Scheme Charge Transfer for Efficient Photocatalytic O2 Evolution Synchronized with Cr(VI) Reduction
Source: Nanomaterials (Basel). 2025 Oct 19;15(20):1592. doi: 10.3390/nano15201592 (PMC12566910; doi:10.3390/nano15201592)
Supplement: Supplementary file 1 [file nanomaterials-15-01592-s001.zip › nanomaterials-3930900-supplementary.pdf]

Supporting information

Interfacial P-O-Cu Bonds Drive Rapid Z-Scheme Charge Transfer for Efficient Photocatalytic O_2 Evolution Synchronized with Cr(VI) Reduction

Yingcong Wei [1], Zeyu Su [1], Bo Weng [2,3,*]

[1] School of Physics and Electronic Engineering, Jiangsu University, Zhenjiang, Jiangsu, 212013, P. R. China

[2] State Key Laboratory of Advanced Environmental Technology, Institute of Urban Environment Chinese Academy of Sciences, 1799 Jimei Road, Xiamen, 361021 P. R. China

[3] University of Chinese Academy of Sciences, 19A Yuquan Road, Beijing, 100049 P. R. China

* Correspondence: bweng@iue.ac.cn

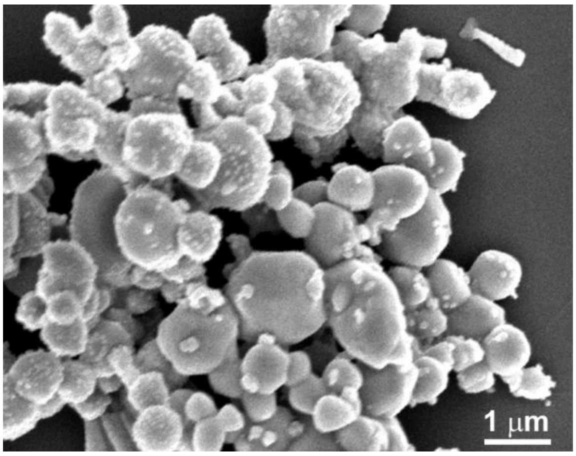

Figure S1. SEM image of Ag_3PO_4.

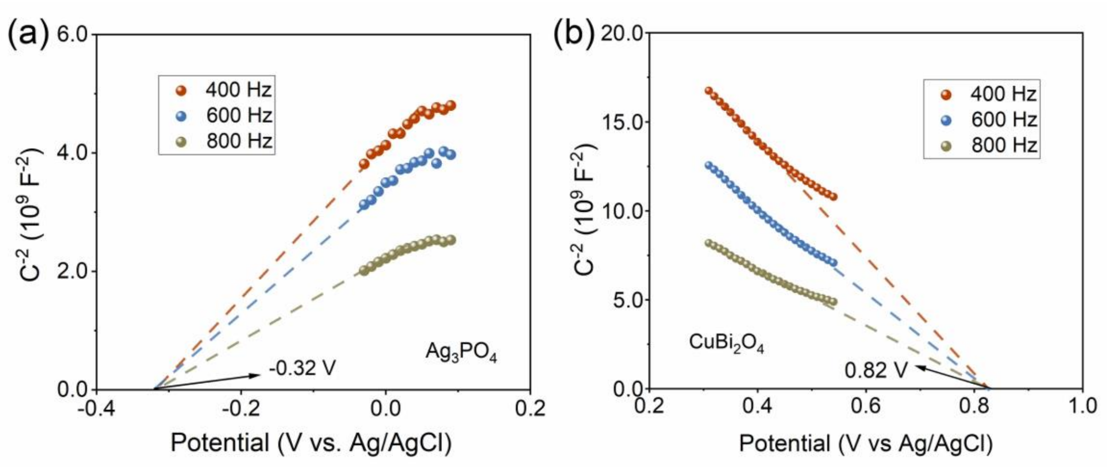

Figure S2. Mott–Schottky (M-S) plot of Ag_3PO_4 (a) and $CuBi_2O_4$ (b).

Table S1. Mass ratio of $CuBi_2O_4$ to Ag_3PO_4 in the composite photocatalyst determined by ICP analysis.

Samples	n_{Bi}/n_{Ag}	$m_{CuBi2O4}/m_{Ag3PO4}$
ACBO-2	0.0072	0.014
ACBO-5	0.0245	0.048
ACBO-7	0.0312	0.061
ACBO-10	0.0455	0.089

Table S2. Comparison of the photocatalytic oxygen evolution performance.

Material Name	Light Wavelength	Oxygen Production Rate ($\mu mol \cdot g^{-1}$)	References
Fe_2O_3	$\lambda \geq 355$ nm	50	Applied Catalysis A: General, 268, pp.159-167.
Ba_2NbO_3N	Visible light	130	Advanced Materials Interfaces, 8, p.2100813.
CoS-2	Visible light	150	Advanced Functional Materials, 27, p.1605846.
Ag_3PO_4/MoS_2 nanocomposites	Visible light	201.6	Frontiers in chemistry, 6, p.123.
Fe-NGO/Ta_3N_5	Visible light	184.7	Chinese Journal of Chemistry, 41, pp.280-286.
ACBO-5	$\lambda \geq 420$ nm	652.7	This work

Table S3. Concentration of Cu, Ag, and Bi ions in solution determined by ICP after photocatalytic reaction.

Ion	Concentration (mmol/mL)
Cu	0.0051
Ag	0.0010
Bi	0.0003
